# Did the notochord evolve from an ancient axial muscle? The axochord hypothesis

**DOI:** 10.1002/bies.201500027

**Published:** 2015-07-14

**Authors:** Thibaut Brunet, Antonella Lauri, Detlev Arendt

**Affiliations:** ^1^ Developmental Biology UnitEuropean Molecular Biology LaboratoryHeidelbergGermany

**Keywords:** axochord, evo‐devo, evolution, mesoderm, musculature, notochord, urbilateria

## Abstract

The origin of the notochord is one of the key remaining mysteries of our evolutionary ancestry. Here, we present a multi‐level comparison of the chordate notochord to the axochord, a paired axial muscle spanning the ventral midline of annelid worms and other invertebrates. At the cellular level, comparative molecular profiling in the marine annelids *P. dumerilii* and *C. teleta* reveals expression of similar, specific gene sets in presumptive axochordal and notochordal cells. These cells also occupy corresponding positions in a conserved anatomical topology and undergo similar morphogenetic movements. At the organ level, a detailed comparison of bilaterian musculatures reveals that most phyla form axochord‐like muscles, suggesting that such a muscle was already present in urbilaterian ancestors. Integrating comparative evidence at the cell and organ level, we propose that the notochord evolved by modification of a ventromedian muscle followed by the assembly of an axial complex supporting swimming in vertebrate ancestors.

## Introduction

The reconstruction of last common ancestors of modern groups is one of the key challenges in evolutionary biology. It is possible by two methods: observation of fossils (which, for early bilaterian evolution, represent a still patchy record 1), and comparison of homologous structures in modern forms, allowing inference of the most likely ancestral states. Homology refers to structures in two modern species that have been inherited from their last common ancestor. It applies at all levels of biological organization – genes, cell types, tissues, and organs.

Regarding notochord evolution, we can thus ask: what structure in the last common bilaterian ancestor gave rise to the chordate notochord? This structure necessarily existed, but its nature and complexity – representing a simple population of cells, a certain tissue, or even a distinct organ such as a specific muscle – remain to be defined. Which structures in non‐chordate lineages has it given rise to? The answers to these questions are currently unclear 2, 3. The notochord has variously been proposed to be related to the stomochord of enteropneusts 4, 5; to the hydrocele of echinoderms 6; to a longitudinal stiffening of the gut 7, 8; and, by one author, to a ventral midline muscle in annelid worms 9. However, none of these homology proposals has gained widespread acceptance.

How can homology of two structures be experimentally tested? The nature of possible supporting evidence is summarized by Remane's triple homology criteria 10, 11: (i) specific quality: similarity in structural detail 12; (ii) position: they should have the same relative position within the body; (iii) continuity: they should be present in phylogenetically intermediate groups. Later, Hennig, building on these criteria 12, emphasized their importance as pre‐requisites for homology (as the cladistics school did after him 13). He extended the continuity criterion by emphasizing the need to test for absence or presence of a character along the branches of a phylogenetic tree to infer ancestral states. According to the cladistic approach, a character is only considered homologous if its distributed presence in a clade supports its likely existence in the last common ancestor. (This method is called “ancestral state reconstruction,” see below.)

We recently published a detailed comparison of cell types between remote groups: those that assemble into a ventromedian muscle in the annelid worms *Platynereis dumerilii* and *Capitella teleta* and those that form the notochord in chordates 14. We found strong similarities between these cells in terms of gene expression, morphogenetic movements, and position in the bodyplan. Following the criteria of structural similarity (i) and topology (ii), our data suggest homology at the cell type level, hence suggesting that the notochordal cells might have arisen from ancient contractile cells in the ventral midline. For obvious reasons, such detailed developmental and molecular investigations have so far only covered few species, and more species need to be examined to test for presence/absence (iii) of these genetic and developmental traits in the bilaterian tree.

**Figure 1:**
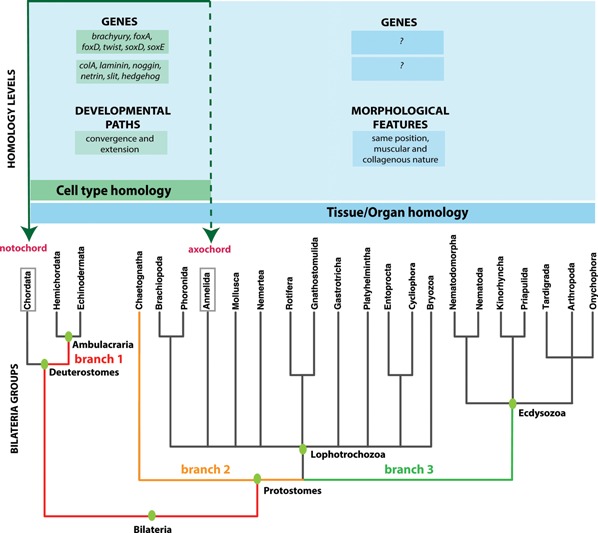
The bilaterian phylogenetic tree, after 15. Levels of homology (cells and organs) are indicated, together with the taxa for which evidence is available as well as the nature of that evidence. The three branches known to separate annelids and chordates are colored. The axochord hypothesis implies conservation of a ventromedian muscle along at least these three branches, and possibly other branches within Lophotrochozoa once their phylogeny is solved.

However, continuity can already be tested at the tissue/organ level, as a vast repertoire of anatomical data is available to test for the presence of ventromedian muscles in various bilaterians (Fig. 1). If a ventromedian muscle were present in the majority of bilaterian lineages, the most parsimonious interpretation according to Hennig's cladistic approach would be that it existed in bilaterian ancestors. As we will outline in the second part of this review, these comparative data make a strong case that such a muscle was indeed present. Future developmental and genetic studies will reveal its cellular characteristics and will allow further testing of the continuity criterion.

## Are the axochord and the notochord homologous?

### Molecular profiling and developmental data support cell type‐Level homology of axochordal and notochordal cells in annelids and chordates

**Figure 2:**
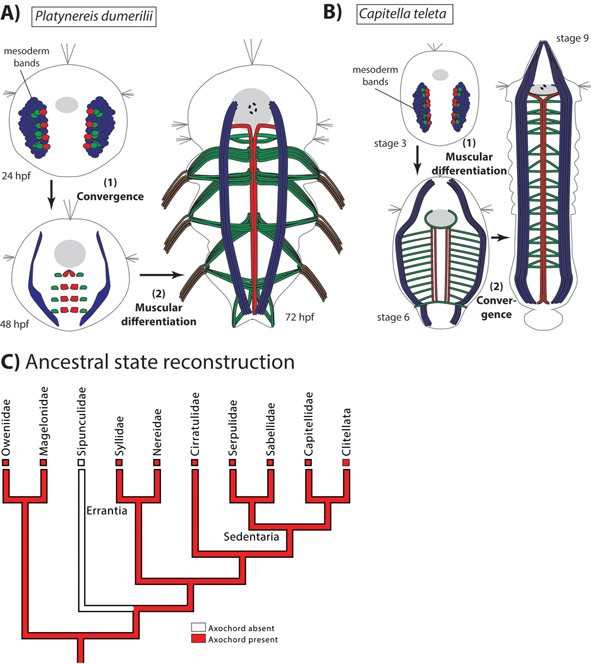
The axochord in annelids. **A:** Development of the axochord in *Platynereis dumerilii* following 14. Red cells are axochordal cells; green cells are presumptive ventral oblique muscles; blue cells give rise to the rest of the mesoderm; foregut is in grey; dotted circle is the mouth. **B:** Development of the axochord in *Capitella teleta* following 14. **C:** Ancestral state reconstruction for annelids. The tree follows 22, 23. Only one group of known phylogenetic position, sipunculids, lacks an axochord (the other family, Sphaerodoridae, has not been included in any phylogenomic analysis).

Similar to the notochord, the *P. dumerilii* axochord develops by convergence‐extension of mesodermal cells towards the midline 14 (Fig. 2A). These cells differentiate into a rod of tissue located between the central nervous system and the axial blood vessel, serve as an attachment band for transverse muscles, and likely secrete a collagen‐rich extracellular matrix (as suggested by the expression of the genes *colA1* and *colA2*). These histological, morphogenetic and positional properties are reminiscent of those of the chordate notochord 16. Moreover, the axochord expresses a specific combination of six transcription factors (*brachyury*, *foxA*, *foxD*, *twist*, *soxD*, *soxE*) and eight effector genes (*colA1*, *colA2*, *chordin*, *noggin*, *netrin*, *slit* and *hedgehog*) that uniquely defines it, and also uniquely define the vertebrate notochord. Together, these 13 genes represent the most complete and most evolutionarily stable molecular profile for notochordal cells that can be put forward after an unbiased screening of the vertebrate literature – thus avoiding arbitrary “cherry‐picking” of markers 17. All the genes of this list that have been investigated in amphioxus are also general notochord markers (apart from *soxE*). Since this combined signature is found nowhere else in the body, its co‐option from another expression territory is unlikely: its convergent acquisition would require multiple, independent and identical co‐option events. This is unparsimonious, since the known instances of convergent cell type evolution have involved the independent production of similar cellular phenotypes by completely different molecular components 18, 19.

It is worth noting that the molecular profile of the axochord includes a notochord‐like combination of signaling molecules (*noggin*, *hedgehog*, *netrin*, and *slit*), suggesting that the annelid axochord, like the notochord, functions as a signaling center. This hypothesis, however, still awaits direct functional testing.

### Bridging the phylogenetic gap between annelids and chordates: A cladistic approach

Obviously, our cellular comparison is powerful with regard to the two first homology criteria (position and specific quality). However, these in‐depth data covered only two annelid genera (*Platynereis* and *Capitella*) – thus leaving unsolved whether the third criterion of continuity (i.e. presence in intermediate groups), is satisfied. Indeed, it has been pointed out that our comparison leaves out a large number of intermediate branches 20. In fact, our current knowledge of the bilaterian phylogeny implies that, if such cells were present in the last common annelid/chordate ancestor, they should also have been present in the ancestors of ambulacrarians (branch 1), chaetognaths (branch 2), and ecdysozoans (branch 3), plus a yet unknown number of branches in the lophotrochozoan stem‐line 21 (Fig. 1). Since (with the exception of the highly specialized fruit flies and nematodes) not much molecular and developmental data are available for these lineages, this hypothesis remains to be tested at the cell type level. Fortunately and interestingly, at the organ level, there is a rich body of comparative anatomical literature covering virtually all bilaterian phyla. As will be outlined below, these comparative data support the ancestral presence of a ventromedian muscle in bilaterians. Future comparative studies will unravel how this ancient ventromedian muscle was genetically specified and how it developed.

## Testing the presence of an axochord across Bilateria

Assessing the presence of a ventromedian muscles from classical morpho‐histological data is challenging, as they are often of insufficient resolution to observe axochord‐like structures, which are frequently of minute size. Facilitating our search, however, a large dataset of phalloidin stainings covering virtually all bilaterian phyla has been produced in the last 20 years, allowing widespread testing for the presence of axochord‐like structures in bilaterians.

### The axochord is conserved across annelids

The first implication of our hypothesis is that the axochord must be an ancestral annelid feature. Annelids are a highly diverse group, for which the internal phylogeny has been recently clarified by phylogenomics 22, 23, making it an ideal test case. Phalloidin stainings have been published for 14 families, covering both main annelid clades (Errantia and Sedentaria) and two families that likely diverged earlier (Oweniidae 14, 24 and Magelonidae 25). Axochord‐like ventromedian muscles have been observed in virtually all of them, and usually serve as attachment bands for transverse muscles. Axochords are always composed of a pair of longitudinal myofibers closely flanking the midline, which contact each other in the main part of the trunk, but diverge at their anterior and posterior extremities (behind the mouth and in front of the anus). The degree of terminal divergence is modest in *Platynereis* and most other genera, but more extensive in *Pomatoceros* 26. In *Prionospio*, both myofibers closely flank the midline, but do not actually touch each other; in this configuration, the corresponding muscle has been called “paramedian muscle” 25.

We hypothesized that the paramedian configuration can be developmentally explained by incomplete convergence toward the midline of axochord‐like precursor cells during early development. We tested this hypothesis by studying an annelid known to possess such a paramedian muscle at early larval stages 27: *C. teleta*, a model species belonging to Sedentaria 22. Phalloidin stainings revealed that the previously documented paramedian muscle fibers converge in late development and form a proper axochord before hatching 14 (Fig. 2B). Axochord development thus underwent a heterochronic shift between *Platynereis* and *Capitella*: in *Capitella*, axochordal cells first form differentiated myofibers and then converge, while in *Platynereis* those events happen in the opposite order (Fig. 2A and B). Gene expression data for all axochord markers investigated (*brachyury*, *foxA*, *netrin*, *slit*, *hedgehog*, and *twist2*) are consistent with expression in the *Capitella* axochord 14, 28, 29. The *Capitella* data thus confirm conservation of at least part of the axochord/notochord molecular signature within annelids, and provide a possible mechanism for the evolutionary transition between paramedian and ventromedian configurations. Finally, in a subgroup of Sedentaria (Clitellata, which include earthworms and leeches), the entire body is surrounded by a continuous longitudinal muscle layer 30, complicating observations. However, in earthworms and leeches, a distinct ventromedian longitudinal muscle (called “epineural muscle” or “capsular muscle” 31, 32) is present immediately above the ventral nerve cord and below the ventral blood vessel – thus representing a bona fide axochord. Like the *Platynereis* axochord, the epineural muscle is firmly embedded within the ventral nerve cord sheath. Its contractions are thought to allow deformation of the nerve cord in concert with body shape changes during peristaltic motion. Molecular data on clitellates are scarce, but the ventromedian myofibers of leeches have been reported to express the specific intermediate filament‐encoding gene *hif‐3*, which is absent from lateral longitudinal muscles 33.

Only two annelid families clearly lack an axochord: Sphaerodoridae 34 and Sipunculidae 35.

The most parsimonious ancestral state for annelids is the presence of a canonical axochord, composed of two adjacent longitudinal myofibers flanking the midline, with attached transverse muscles (Fig. 2C). Importantly, conservation of a stereotypical axochord is compatible with the huge variety of annelid lifestyles and morphologies, including sessile suspension‐feeders, errant bottom‐dwellers, burrowers, and undulatory swimmers.

Conclusions about other phyla face two main limitations: for most, the internal phylogeny is still under debate (apart from annelids, molluscs, arthropods, and chordates), and the interrelationships of the phyla themselves (i.e. the higher‐order bilaterian phylogeny) remain partially unresolved. While “Chordata,” “Ambulacraria,” “Ecdysozoa,” and “Spiralia” seem stable, their internal branching is more contentious. Moreover, the bilaterian phylogeny is strongly dichotomous: the general structure of the animal phylogenetic tree seems closer to successive symmetrical bifurcations between equally large groups, than to successive branching of individual phyla from one stem – hence producing a “balanced” or “symmetrical” phylogenetic tree 36, 37. In such a tree, there are no strategically located “basal” branches that would carry higher weight on the inferred ancestral states at key nodes, and conclusions can only be reached after examination of a broad sample. With these caveats in mind, a survey of the available data allows some insights into musculature evolution and the possible ancestrality of ventromedian muscles.

### The axochord is conserved across non‐Annelid spiralians

Annelids are part of the superphylum Spiralia, which includes both large coelomate animals and small acoelomate groups of the interstitial fauna (or “platyzoa,” which are likely not monophyletic 21) 38. One additional microscopic phylum, Cycliophora, exclusively lives as a commensal on the mouthparts of lobsters 39. Strikingly, axochord‐like ventromedian muscles have been described in both coelomate and acoelomate spiralians. For ancestral state reconstruction, we will use a recent phylogeny of Spiralia 21, which proposes that this clade is composed of three monophyletic groups: Lophotrochozoa (containing all coelomate spiralians), and two acoelomate groups: Rouphozoa and Gnathifera.

#### An axochord is present in molluscs, brachiopods and nemerteans

**Figure 3:**
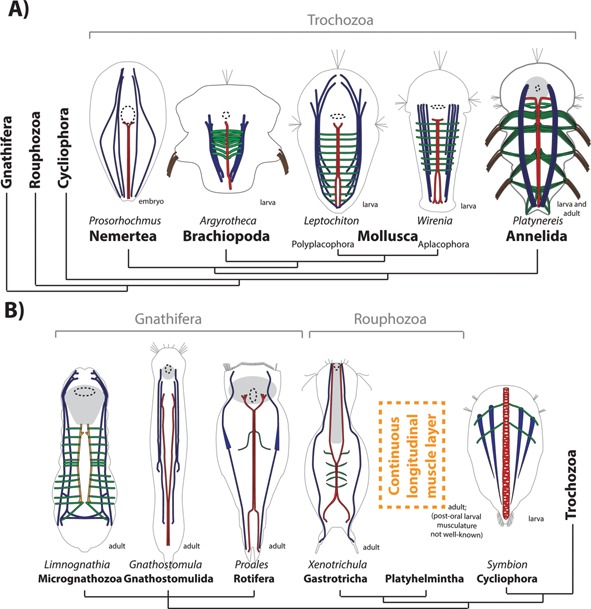
The axochord in spiralians. The tree follows 21. **A:** Trochozoa. Annelid after 14, molluscs after 40, brachiopod after 43 (and personal communication of Dr. Andreas Altenburger), nemertean after 46. **B:** Cycliophora, Rouphozoa and Gnathifera. Cycliophoran after 39, gastrotrich after 54, rotifer after 49 (note that the schematic in this paper presents the paired ventromedian fibers slightly more distant than they are in the actual specimen; Dr. Martin V. Sørensen, personal communication), gnathostomulid after 52, micrognathozoan after 53. Colors are as in Fig. 2A, and the paramedian muscle of micrognathozoans is orange.

In molluscs, a ventromedian muscle composed of adjacent paired fibers has been described in the larvae of Aplacaphora (*Wirenia argentea*) and Polyplacophora (*Leptochiton asellus* and *Mopalia muscosa*) 40 (Fig. 3A), where it serves as an attachment point for transverse muscles. Together, Aplacophora and Monoplacophora form a clade considered the sister‐group of all other molluscs 41, 42. The ventromedian and transverse muscles exist only transitorily during mollusc development, and have been speculated to represent recapitulative instances of ancient structures 40.

In brachiopods, ventromedian myofibers have been detected in the ventral midline of the early three‐lobed larvae of *Argyrotheca* and *Terebratalia* (43 and Dr. Andreas Altenburger, personal communication; Fig. 3A and Supp. Fig. S1). Conserved expression of the annelid axochord markers *mox*, *foxD*, and *noggin* has been reported in a stripe of ventromedian mesoderm in *Terebratalia*, suggesting conservation of the axochord molecular profile between annelids and brachiopods 44. At very late larval stages, only faint phalloidin stainings are visible in the ventral midline 44, 45, which suggests the *Terebratalia* axochord might grow at a smaller rate (or regress) compared to other ventral muscles; its earlier presence is however unambiguous (43 and Fig. S1).

In nemerteans, a ventromedian muscle (without transverse fibers) is among the first muscles to form in the embryo of *Prosorhochmus* 46 (Fig. 3A).

One acoelomate phylum has been tentatively assigned to Lophotrochozoa 15: the minute cycliophorans. In this group, planktonic larvae possess a hugely expanded and vacuolized ventromedian muscle: the “chordoid organ” 39, 47, 48 (Fig. 3B). Its function in a larva that moves primarily by ciliary beating is unclear: its role might be maintaining body shape (as both the axochord and the notochord do 14), and in particular bracing the midline when ventrolateral muscles contract during turning. The chordoid organ cells contain circular myofilaments, organized as “ring fibers” surrounding the vacuoles. The peculiar orientation of these fibers might be a consequence of vacuolization (see below).

Ectoprocts and entoprocts, which lack unambiguous dorsal and ventral sides, are not considered here, as their strongly modified bodyplan precludes comparisons.

#### Axochords have a mosaic presence in gnathiferans (rotifers, gnathostomulids and micrognathozoans) and rouphozoans (platyhelminthes and gastrotrichs)

In interstitial phyla, paired myofibers closely flanking the ventral midline have also been reported – for example, in the trunk of the rotifers *Proales daphnicola* 49 and *Brachionus urceolaris* 50, of the gastotrich *Xenotrichula intermedia* 51, and of the gnathostomulid *Gnathostomula peregrina* 52 (Fig. 3B). As in annelids, they diverge anteriorly and posteriorly, and some other species display the same fibers in a more divergent, paramedian configuration. Variable degrees of convergence (ranging all the way from ventromedian to paramedian) can coexist within the same genus – for example, *Proales* (rotifer) 49, or *Xenotrichula* (gastrotrich) 51. Despite these differences, this muscle has been recognized as clearly being the same under both configurations (from its position, general morphology and connections) in the descriptions of these genera. This suggests that, as in annelids, the transition between ventromedian and paramedian muscles is easily achieved by complete versus partial convergence processes. The adaptive significance for these varying degrees of convergence is unclear.

In *Limnognathia maerski*, the only species of the small gnathiferan phyla Micrognathozoa, transverse muscles are attached to a paramedian muscle, which itself is attached to the posterior border of the pharynx 53 (Fig. 3B) – hence displaying connection properties similar to the ventromedian/paramedian muscles of other gnathifers and of annelids. We hypothesize here that there is homology between those midline‐flanking paired longitudinal muscles across Spiralia: while varying degrees of convergence can result in slightly different morphologies, their connection properties are conserved, as their molecular profiles should be – allowing eventual testing of this hypothesis by expression profiling.

#### The axochord likely represents an ancestral spiralian feature

An annelid‐like axochord has been reported for the majority of spiralian phyla, and usually serves as an attachment band for repeated transverse muscles. The fact that the axochord is a sometimes transient feature of early development supports its ancestral presence in Spiralia and argues for evolutionary transitions from ancestral muscular systems based on antagonism between ventromedian, transverse, and ventrolateral myofibers (possibly already surrounded by a circular layer 55), to worm‐shaped peristaltic forms relying exclusively on continuous longitudinal and circular layers (e.g. the adults of some large nemerteans), or to sessile lophophorate forms (e.g. adult brachiopods). A temporary embryonic/larval axochord might persist by sheer phylogenetic inertia, or it might still fulfill transient function, such as larval locomotion or signaling.

### Is an axochord conserved in Ecdysozoa?

The internal ecdysozoan phylogeny is still unclear 48, 49, 50, 56, 57. Three frequently proposed clades are Panarthropoda (onychophorans, tardigrades, and arthropods), Scalidophora (priapulids, kinorhynchs, and loriciferans), and Nematoida (nematodes and nematomorphs), and we follow this view here. Ecdysozoans are defined by the shared presence of a moulting exoskeleton 58 which, in several phyla, shows a tendency to become increasingly rigid and to replace muscles as supporting structures or as antagonists. Some degree of repeated muscle loss would thus be unsurprising in ecdysozoans. Nevertheless, some phyla have an axochord, and hypotheses on the evolution of ecdysozoan musculature can be proposed.

#### Ventromedian muscles are present in Scalidophora (kinorhynchs and loriciferans)

**Figure 4:**
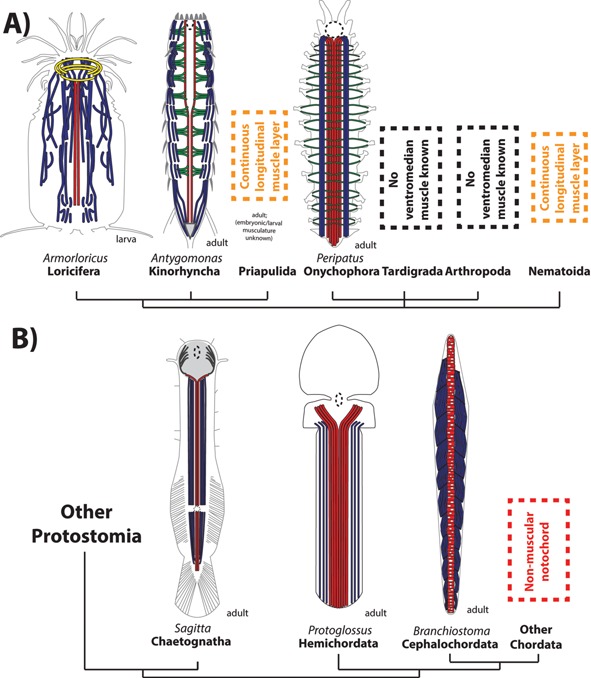
The axochord in ecdysozoans and deuterostomes. **A:** Ecdysozoans. Loriciferan after 60, kinorhynch after 59. Onychophoran reconstituted after 62. **B:** Deuterostomes and chaetognaths. Chaetognath after 14. Hemichordate reconstituted after after 77. Colors are as in Fig. 2A.

Paired ventromedian muscles serving as an attachment band for transverse muscles exist in the kinorhynch *Antygomonas* 59 and in the Higgins larva of the loriciferan *Armorloricus* 60 (Fig. 4A). Adult priapulids rely on antagonism between continuous longitudinal and circular layers around the body, as typical for burrowing worms 30, and the musculature of embryonic/larval priapulids is still incompletely known (but see Ref. 61 for a recent description of the *Priapulus caudatus* larval musculature with a mention of a ventromedian retractor muscle in the first lorica larva).

#### Ventromedian muscles are present in onychophorans but not in tardigrades

Onychophorans have a hugely developed ventromedian muscle 62 (Fig. 4A) – which probably acts in bracing the body, and notably in preventing deformation of the ventral side (housing the ventral nerve cord) during hydrostatic expansion/retraction of lateral appendages. Unlike the annelid axochord, the onychophoran ventromedian muscle is not an attachment band for transverse muscles: onychophoran appendage muscles attach to the tegument, on a structure called the ventral organ 63. This argues against homology of onychophoran appendicular muscles to the annelid ventral oblique muscles moving the parapodia – consistent with the common assumption that Urbilateria lacked trunk appendages 64, and that different muscles might have been co‐opted or neoformed for appendage movement in different phyla. No ventromedian muscle is known in the tardigrade trunk (though a minute one is present in the foregut 65, 66).

#### The special case of the arthropod mesodermal midline glia: a modified axochord?

In line with the evolution of a sclerotized cuticle as a supporting scaffold, arthropods have been proposed to have undergone a massive reduction of their ancestral onychophoran‐like circular/longitudinal musculature, that lost its ancestral bracing function 67. Any ventromedian muscle (absent from all investigated arthropods) would plausibly have been lost in this process.

However, insects do possess a non‐muscular mesodermal midline: the so‐called “mesodermal midline glia” or “DM cells” (“dorsal median,” as they are positioned immediately dorsally to the central nervous system). At first sight, the *Drosophila* mesodermal midline glia seem to display a number of similarities to the axochord: they are present under the form of segmentally repeated pairs of cells immediately below the ventral nerve cord 68; are required for commissural axon guidance 69, express *netrin* 70, and are specialized in matrix secretion 71 – including some common components with axochord and notochord (laminin) but also some that are not (collagen IV and the arthropod‐specific protein glutactin). Finally, the key defining transcription factor of the DM cells, the homeodomain protein *mox/buttonless* 69, is also expressed in the *Platynereis* axochord (but not in the notochord) 14.

However, a number of key differences cast doubt on the homology of the mesodermal midline glia to the axochord: 1) DM cells, which have an elongated monopolar shape, extend long lateral processes in a transverse, rather than longitudinal, direction 68, 69 2) DM cells coexpress *paraxis* (*CG12648*/*CG33557*) 72 and *engrailed* 73, which together represent a specific profile for the annelid ventral oblique muscles – which also express *mox* and *netrin* 14. On the other hand, DM cells express none of the specific axochordal/notochordal transcription factors (such as *brachyury* and *foxA*). 3) The lateral processes of DM cells are anchored at the attachment point of the lateral longitudinal muscles on the body wall (muscle 7) 68. These connection properties are expected if they are equivalent to transverse muscles, which in annelids reach out to the ventrolateral longitudinal muscles (Fig. 2A) – but have nothing to do with those of ventromedian myocytes.

By their molecular profile, orientation and muscular connections, DM cells are more similar to annelid ventral oblique muscles than to the axochord, and it can be hypothesized that they are modified transverse muscles. In this hypothesis, if a ventromedian muscle was ancestrally present in panarthropods (as suggested by the onychophoran situation), it would have been entirely lost in *Drosophila*, and former transverse myocytes would have come to occupy the vacant mesodermal midline. This homology hypothesis is testable in several ways: while, as noted above, onychophorans lack annelid‐like transverse muscles, they might possess *mox/en/netrin/paraxis*+ DM‐like cells – which should attach both to the ventromedian muscle (lost in *Drosophila*) and to the lateral longitudinal muscles (as in *Drosophila*); the transverse muscles of kinorhynchs would be interesting to investigate in this respect.

#### The axochord has most likely been lost in Nematoida (nematodes and nematomorphs)

Nematoids have a near‐continuous longitudinal muscle layer surrounding the body. Unlike the clitellate configuration, this longitudinal muscle layer lies internal to the ventral nerve cord (though the nervous ganglia secondarily “sink” below the muscle layer by crossing it during nematomorph development 74). Conservation of the axochord molecular profile in nematoids is unlikely, because, at least in the model nematode *C. elegans*, several key axochord/notochord genes (including *brachyury*, *colA*, and *soxE*) have simply been lost from the genome. This makes it difficult to identify any potential axochord homolog, which might either have been lost or modified beyond recognition. Ventral longitudinal muscles of *C. elegans* still specifically express *unc130*/*foxD* 75 and *netrin* 76 (two ventral somatic muscle markers in *Platynereis*), but not *foxA* (*PHA‐4*) – showing that, while some general musculature patterning is recognizable in nematodes, a specific axochord homolog cannot be identified. The axochord might thus have been lost, together with transverse muscles, in conjunction with the evolution of the specialized nematoid locomotion, relying on the antagonism between ventro‐ and dorsolateral muscle blocks and an elastic cuticle 30.

#### The axochord has a mosaic presence in ecdysozoans

Of the three ecdysozoan clades, only scalidophorans can be inferred to ancestrally possess an axochord. The panarthropod ancestral state is undetermined: only onychophorans have a clear ventromedian muscle. Finally, nematoids possess a simplified musculature – and, at least in *C. elegans*, a simplified genome. The ancestral state for ecdysozoans therefore remains undecided. However, the clear presence of an axochord in at least three ecdysozoan phyla – and its inferred ancestral presence in the outgroups Spiralia and Chaetognatha (see below) – make the hypothesis of an ancestral ecdysozoan axochord attractive. To this ground pattern, scalidophoran‐like transverse muscles might be added. Molecular characterization of ecdysozoan ventral mesodermal cells will be key in testing these hypotheses.

### A ventromedian muscle is present in Chaetognatha, a possible protostome outgroup

Chaetognatha is a relatively small (but very abundant) phylum (120 species) of worm‐shaped swimming invertebrates, which might have diverged before all other protostomes 78, 79. The chaetognath body comprises pairs of coelomic cavities surrounding the gut, separated along the midline by myoepithelial dorsal and ventral mesenteries 80. The body is almost entirely surrounded by strong longitudinal striated muscles. Flanking the ventral midline, directly connected to the ventral mesentery, are specialized longitudinal myofibers of triangular cross‐section, which present a unique type of striation, and are hence called “secondary muscles” 81. This distinguishes them from all neighboring ventrolateral longitudinal muscles, as this peculiar striation type is only present in two other locations in the body (laterally and in the dorsal midline). Their nature, orientation, triangular shape and connection to the ventral mesentery are reminiscent of the axochord. Moreover, like the axochord, the chaetognath ventromedian longitudinal muscle bifurcates behind the foregut 14 (Fig. 4B). The chaetognath ventromedian muscle might thus be an axochord homolog.

No transverse muscles are known. The weak circular smooth fibers of myoepithelial cells within the mesenteries provide some limited antagonism to longitudinal muscles, but they are incomparable in nature and position to the transverse muscles of other protostomes 80. Transverse fibers might have been lost during the evolution of the highly specialized chaetognath undulatory swimming, which is effected by dorso‐ventral antagonism; alternatively, they might have evolved only after chaetognaths branched off the protostome stem.

## Is the axochord an ancient deuterostome feature?

The deuterostome tree is a bifurcation between Ambulacraria (echinoderms and hemichordates, plus potentially xenacoelomorphs), and Chordata.

### Ventromedian muscles have a mosaic presence in Ambulacraria (hemichordates and echinoderms)

Hemichordates include the worm‐like enteropneusts and the sessile suspension‐feeding pterobranchs. Enteropneusts might be paraphyletic 82, or both groups might be monophyletic 82, 83. Most authors have argued so far for the enteropneust morphology being closer to the ancestral situation 84, 85, and we follow this view here. The most popular candidate for a notochord homolog in enteropneusts has historically been the stomochord, an anterior vacuolated expansion of the pharynx into the proboscis 4, 5. Its very anterior and dorsal position argues against any affinity to the notochord; moreover, it lacks expression of key notochord/axochord markers such as *brachyury, foxA*, and *noggin* 86, 87 – but it does express *colA*, in line with its structural role 88. While the stomochord underlies the invaginating neural cord of the collar, thus displaying morphological similarities to the chordate notochord/neural tube complex 89, and expresses *hedgehog* 88, the bulk of molecular and anatomical data argue against its homology to the notochord. Expression of *nk2.1* and *foxE* suggests instead affinities to part of the chordate foregut – possibly the endostyle/thyroid 87, 90.

Since morphological 91 and molecular data 92 suggest that hemichordates, unlike chordates, are not dorsoventrally inverted compared to protostomes, a genuine notochord homolog should be looked for in the ventral trunk. Such a candidate structure is the pygochord, a vacuolated thickening of the ventral mesentery of ptychoderid enteropneusts. However, the pygochord is located between the ventral blood vessel and the gut – unlike the axochord and the notochord, which are positioned between the axial blood vessel and the central nervous system. The best candidate for a notochord/axochord homolog would be a striated paired ventromedian muscle between the ventral nerve cord and the ventral blood vessel.

The enteropneust trunk is almost entirely surrounded by longitudinal myofibers. While no enteropneust phalloidin staining has been published (apart from the interstitial species *Meioglossus* 93), histological data indicate the existence of conspicuous paired ventromedian fibers connected to the ventral mesentery in *Protoglossus* (Fig. 4B) 77, 94, and of smaller similar fibers in *Saccoglossus* (95 and Dr. Sabrina Kaul‐Strehlow, personal communication) – so small that they are usually omitted from classical schematics 96. In *Protoglossus*, they diverge behind the foregut 77. However, the ventromedian fibers display no reported morpho‐anatomical feature that would readily distinguish them from ventrolateral longitudinal muscles. Molecular individuality might nevertheless exist, as suggested by the specific expression of the transcription factors *mox* and *foxD* in the ventral‐most mesodermal cells of the developing *Saccoglossus* – the location from which the ventromedian myofibers should originate 92, 97. Again, more developmental and molecular studies will be needed to assess further the potential existence of a hemichordate axochord homolog.

Echinoderms display a highly modified adult bodyplan 30, and their early larvae only possess visceral muscles 98. Electron microscopy has provided hints to the presence of a more complex somatic muscle system in late starfish bippinaria larvae 99, but this system still awaits characterization by phalloidin stainings. With the data at hand, it is reasonable to assume that the ventromedian mesoderm has most likely been lost in echinoderms, or modified beyond recognition.

### What is the origin of the chordate notochord and backbone?

#### Did an axochord evolve into the notochord?

Finally, we propose that dorsoventral inversion in the stem lineage of chordates put the former axochord in a dorsal position. The muscular notochord of amphioxus 100, 101 would represent a clue to this transition (Fig. 4B). Evolution of the notochord from a paired median muscle would be consistent with the fact that, after it forms by evagination from the archenteron roof, the early amphioxus notochord is composed of two adjacent longitudinal rows of cells, which secondarily intercalate into a single series (“stack of coins”) 102. In conjunction with increased reliance on undulatory swimming, the notochord acquired incompressible intracellular vacuoles 103, hence preventing shortening and making it an elastic antagonist. In amphioxus, contraction of the myofilaments fine‐tunes the notochord stiffness during locomotion 104. The appearance of such vacuoles would have entailed a new distribution of mechanical constraints within the notochordal cells, resulting in realignment of the myofilaments in a transverse direction. Consistently, vertebrate longitudinal muscles have been observed to develop unusually oriented filaments, perpendicular to their main axis (“ring fibers”), in response to membrane buckling due to hypercontraction 105 or in myopathies with pathological vacuolization 106.

The ancestral axochord was likely present along the whole trunk and bifurcated behind the mouth, as observed in modern protostomes and enteropneusts (see above). In chordates, the notochord is also present along the whole length of the trunk, but does not bifurcate behind the mouth 30 – consistently with the idea that chordates secondarily evolved a new mouth, non‐homologous to the ancestral bilaterian mouth 107. The loss of the ancestral mouth might have allowed some plasticity in the antero‐posterior extension of the notochord, which reaches the anterior tip of the animal in amphioxus, but is restricted to the tail in tunicates and stops behind the infundibulum in vertebrates 8.

Unlike the annelid axochord, the notochord never presents any overt morphological segmentation. This non‐segmented character was considered significant enough in the early 20th century to constitute a fatal objection to the hypothetical derivation of chordates from annelid‐like ancestors 2. Today, this objection seems less serious, as it is more broadly accepted that segmentation of a structure can be lost or gained during evolution. For example, the lateral plate forms segmentally in amphioxus, but is unsegmented in vertebrates 8; still, its homology is undisputed. Nevertheless, it is worth noting that the frog notochord has been reported to secrete extracellular matrix in a segmental fashion 108, 109.

#### Did the transverse muscles evolve into pioneer myocytes?

In both annelids and chordates, the axochord/notochord acts as an attachment band for lateral locomotory muscles that develop directly adjacent to it. In annelids, they are called “ventral oblique muscles”. In zebrafish, the only muscle cells directly contacting the notochord (“pioneer myocytes”) develop from the paraxial mesoderm cells that are closest to the chordamesoderm (adaxial cells). In both annelids and chordates, these muscles are uniquely defined by coexpression of *foxd* and *engrailed* 14, 110, suggesting homology of the annelid ventral oblique muscles to the chordate pioneer myocytes.

**Figure 5:**
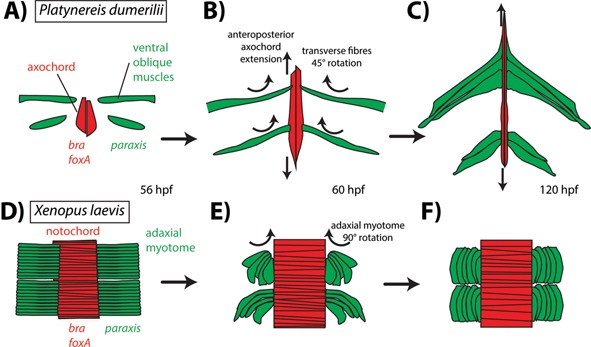
Rotation of transverse myofibers in early development of Platynereis and Xenopus. Axochord/notochord in red, transverse myofibers in green. **A:** 45° rotation of the developing transverse myofibers in Platynereis, forming ventral oblique muscles, drawn after 116. **B:** 90° rotation of adaxial myofibers in Xenopus, forming longitudinal muscles, drawn after 113.

The orientation of these muscles, however, differs between both phyla – transverse in annelids, longitudinal in chordates. Indeed, according to one hypothesis, transverse muscles have been lost in chordate ancestors, after having been replaced by the elastic notochord as antagonists to the longitudinal musculature 111. Interestingly, in frogs, the early slow myofibers arising from adaxial cells develop in a transverse orientation – and only reorient later to become longitudinal 112 (Fig. 5B). This reorientation has been proposed to underlie, at least in part, the “somite rotation” affecting the *Xenopus* myotome 113, 114, 115. The *Platynereis* ventral oblique muscles undergo a more limited 45° rotation, from an initially transverse orientation to the “oblique” direction that gave them their name (Fig. 5A). In vertebrates, the axial complex composed of notochord and attached longitudinal muscles has been complemented by the evolution of a new structure: the rigid backbone (see Box 1).

Box 1 **The origin of the sclerotome**

After having shifted from muscular‐contractile to chordoid‐supporting functions, the notochord formed (together with the cartilaginous gill slits and oral cirri 117, 118, 119, 120) the first chordate skeleton. The notochord was later complemented (and in some species, like ourselves, almost entirely replaced) by the backbone, in the form of first cartilaginous – and later mineralized – vertebrae. The origin of the backbone remains mysterious, but clues might come from comparative data within deuterostomes. The amphioxus sclerocoel develops as an evagination of the coelomic lining facing the future dorsal aorta (the dorsal mesentery), that expands and folds dorsally to surround the notochord and the neural tube 96, 121, 122, 123 (see Fig. C; but see Ref. 124 for a slightly different view). The amphioxus sclerocoel has been argued, from morphological data, to be a sclerotome homolog 122, but this still awaits molecular confirmation. The simplest vertebrae‐like structures are known in hagfish, as small cartilaginous nodules around the dorsal aorta, on the inner side of the dorsal mesentery 125. Similarly, in lamprey, the first sclerotome develops as a thin line of *Col2α1a*+ somitic cells ventral to the hypochord, bordering the space in which the future dorsal aorta will form 126; the sclerotome then expands dorsally, as the amphioxus sclerocoel, to form an extensive axial skeleton surrounding the notochord and the neural tube. Finally, in vertebrates, the sclerotome still gives rise to both vertebral tissue and to smooth myocytes in the wall of the dorsal aorta (Fig. D) 127, 128, 129 – which are proposed to represent remnants of the ancestral lining of the dorsal aorta (the dorsal mesentery) before the vascular endothelium evolved 130.

Taken together, these data suggest that the sclerotome evolved from a local thickening of the dorsal mesentery (Figure). This localized supporting structure would have ancestrally provided a ventral support to the notochord, and then secondarily formed the vertebral centra by expanding around the notochord; at intersegmental septa, dorsal expansion gave rise to dorsal arches and ventral expansion to haemal arches, providing the basic groundplan for a vertebra 8. Fusion of primitive vertebral tissue from adjacent somites lying on both sides of the septum further stiffened the skeleton, and this process is still recapitulated nowadays during vertebrate development by sclerotome resegmentation 8, 131.

In enteropneusts as well, the ventral mesentery (homologous to the chordate dorsal mesentery) has at least once been modified into a skeletal structure: the pygochord of ptychoderid enteropneusts is a stiffened thickening of the ventral mesentery immediately continuous with the lining of the ventral blood vessel (Fig. B) 132. There thus seems to be a tendency in deuterostomes for modifying the ventral mesentery (dorsal in chordates) into a supporting organ. Pygochord and sclerotome would thus illustrate an instance of parallel evolution, i.e. of independent, similar modification of the same ancestral organ in two sister groups 133. Future research will determine whether the ancestral ventral mesentery already performed discrete and hitherto unrecognized supporting functions, paving the way for its further modification into pygochord and sclerotome.

**Figure.** Origin of the sclerotome from the deuterostome axial mesentery. **A:** Cross‐section of the Platynereis trunk, showing connexion of the axochord to the ventral mesentery, the hollowing of which forms the ventral blood vessel. Drawn from 14 **B:** Cross‐section of the Ptychodera trunk, which features a connexion between the ventral longitudinal muscle mass and the ventral mesentery housing the blood vessel. Just dorsal to the blood vessel, a vacuolated expansion of the mesentery forms the pygochord. Drawn from Ref. 134 **C:** Development of the amphioxus sclerocoel from the early dorsal mesentery, outlining the paired cephalochordate dorsal aorta. Drawn from 122. **D:** Development of the vertebrate sclerotome from the ventrolateral lining of the epithelial somites. The sclerotome gives rise to vertebrae and to smooth myocytes around the dorsal aorta. Note the fusion of the early paired dorsal aorta into a unique median vessel. Drawings are according to published cross‐sections of mammalian embryos: for the first two panels, humans 135, 136 and for the third one, pig 137.
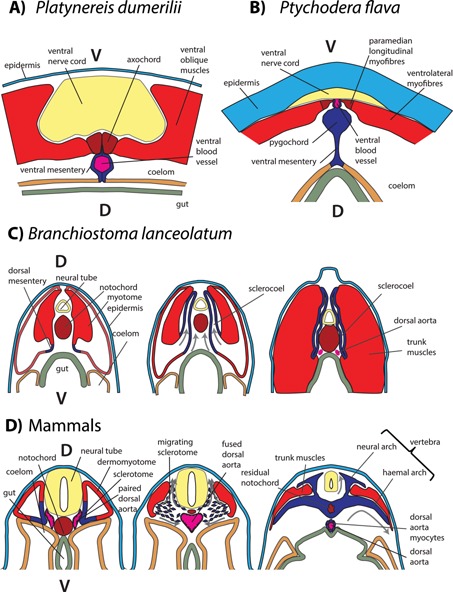

## Conclusions and outlook

Emerging comparative molecular and morphological data make it possible, for the first time, to propose a groundplan for the ventral musculature of the last common bilaterian ancestor – including a ventromedian muscle, transverse muscles attached to it, and paired ventrolateral muscles. Morphological data suggest that a circular muscle layer around the body could be added 55. A very similar plan can still be recognized in some groups, such as polychaetes (like *Platynereis*), kinorhynchs, and larval molluscs and brachiopods (all of which, however, lost the circular musculature). We hypothesize that this groundplan was still present, in a largely unmodified fashion, in the last common ancestors of Spiralia and Ecdysozoa – while the last common ancestor of Deuterostomia might have already lost (or radically modified) the transverse muscles, as they are absent from all living representatives. By vacuolization, the ventromedian muscle gave rise to the chordate notochord, which came to lie dorsally after dorsoventral inversion. A rigorous ancestral state reconstruction based on morphological data supports the presence of a ventromedian muscle in bilaterian ancestors 14. These converging and mutually supportive evidence from comparative anatomy (covering many phyla) and molecular developmental biology (from vertebrates and annelids) make the axochord hypothesis a plausible and stimulating explanation for the origin of the chordate notochord. However, alternative hypotheses cannot yet be ruled out: for example, the axochord and the notochord could have evolved independently from mesenchymal cells, which would have acquired contractility separately in the protostome and deuterostome lineages. Cell type‐level comparisons between a broader range of bilaterian phyla will help elucidating this issue. In the future, better resolution of the bilaterian tree and extension of the molecular and developmental studies to more groups will be key to further assessing these hypotheses.

## Supporting information

As a service to our authors and readers, this journal provides supporting information supplied by the authors. Such materials are peer reviewed and may be re‐organized for online delivery, but are not copy‐edited or typeset. Technical support issues arising from supporting information (other than missing files) should be addressed to the authors.

**Figure S1**. Structure of the brachiopod axochord. **A:** Z‐projection of confocal stack of an early Terebratalia transversa three‐lobed larva with stained nuclei (DAPI) and musculature (phalloidin). Ventral view, anterior side up. Body outline in thin white dotted line. Mouth is thick white dotted circle. Newly generated projection from a stack generously provided by Dr. Andreas Altenburger and previously mentioned in [43]. **B:** Schematic drawing of the same individual. As the paired nature of the axochord is unclear in observations, it is indicated with dotted line.
