# Supplementary material for: Did the notochord evolve from an ancient axial muscle? The axochord hypothesis
Source: Bioessays. 2015 Jul 14;37(8):836–50. doi: 10.1002/bies.201500027 (PMC5054868; doi:10.1002/bies.201500027)
Supplement: Supplementary file 1 — Figure S1. Structure of the brachiopod axochord. A: Z‐projection of confocal stack of an early Terebratalia transversa three‐lobed larva with stained nuclei (DAPI) and musculature (phalloidin). Ventral view, anterior side up. Body outline in thin white dotted line. Mouth is thick white dotted circle. Newly generated projection from a stack generously provided by Dr. Andreas Altenburger and previously mentioned in [43]. B: Schematic drawing of the same individual. As the paired nature of the axochord is unclear in observations, it is indicated with dotted line. [file BIES-37-836-s001.docx]

***Supplementary Figure 1.*** *Structure of the brachiopod axochord*. ***A:*** *Z-projection of confocal stack of an early* Terebratalia transversa *three-lobed larva with stained nuclei (DAPI) and musculature (phalloidin). Ventral view, anterior side up. Body outline in thin white dotted line. Mouth is thick white dotted circle. Newly generated projection from a stack generously provided by Dr. Andreas Altenburger and previously mentioned in [43].* ***B:*** *Schematic drawing of the same individual. As the paired nature of the axochord is unclear in observations, it is indicated with dotted line.*
